# Active Ingredients from *Euodia ruticarpa* Steam Distilled Essential Oil Inhibit PC-3 Prostate Cancer Cell Growth via Direct Action and Indirect Immune Cells Conditioned Media In Vitro

**DOI:** 10.3390/cimb43020071

**Published:** 2021-08-28

**Authors:** Tzu-He Yeh, Jin-Yuarn Lin

**Affiliations:** Department of Food Science and Biotechnology, National Chung Hsing University, 250 Kuo-Kuang Road, Taichung 40227, Taiwan; http00000@yahoo.com.tw

**Keywords:** *Euodia ruticarpa*, PC-3 prostate cancer cell, splenocyte-conditioned media, steam distilled essential oil, Th1/Th2 cytokines

## Abstract

Active constituents isolated from *Euodia ruticarpa* (ER) steam distilled essential oil (SDEO) against PC-3 prostate cancer cell growth remain unclear. To clarify the puzzle, ER SDEO was extracted and further resolved into six isolated fractions ERF1–F6 with Sephadex LH-20 gel filtration chromatography to analyze their biological activities. Active ingredients in the isolated fractions were analyzed with GC-MS. Potential isolated fractions were selected to treat PC-3 cells with direct action and indirect treatment by mouse splenocyte- (SCM) and macrophage-conditioned media (MCM). The relationship between PC-3 cell viabilities and corresponding total polyphenols, flavonoid contents as well as Th1/Th2 cytokine profiles in SCM was analyzed using the Pearson product–moment correlation coefficient (r). As a result, ERF1–F3 was abundant in total polyphenols and flavonoids contents with diverse active ingredients. Treatments with ERF1–F3 at appropriate concentrations more or less inhibit PC-3 cell growth in a direct action manner. Only SCM, respectively, cultured with ER SDEO and ERF1–F3 markedly enhanced the effects to inhibit PC-3 cell growth, suggesting that secretions by splenocytes might involve anti-PC-3 effects. There are significantly negative correlations between PC-3 cell viabilities and IL-2, IL-10 as well as IL-10/IL-2 ratios in the corresponding SCM. Total polyphenol and flavonoid contents in the media cultured with ER SDEO isolated fractions positively correlated with IL-10 (Th2) and IL-10/IL-2 (Th2/Th1) cytokine secretion ratios by splenocytes, indicating that polyphenol and flavonoid components in ER SDEO isolated fractions promote Th2-polarized and anti-inflammatory characteristics. These new findings concluded that the inhibitory effects against PC-3 prostate cancer cell growth are attributed to active anti-inflammatory ingredients in ER SDEO and its active ERF1–F3 fractions through direct action and indirect treatment by modulating splenocytes’ cytokine secretion profiles.

## 1. Background

Prostate cancer is a heterogeneous malignant tumor that occurs in elderly men, mostly resulting from age, diet, hormones and genetics [1]. To date, treatments for prostate cancers include castration therapy, radiation therapy, androgen deprivation therapy (ADT), chemotherapy and possible immunotherapy [2]. However, testosterone, a hormone produced in the testicles, may further stimulate prostate hyperplasia in some patients [3]. Most advanced prostate cancer patients evolve an uncontrollable castration-resistant prostate cancer (CRPC), resulting in scantiness of hormones in the body and increasing the difficulty for prostate cancer treatments [4]. Therefore, it is more and more important to research a new and curative treatment program for prostate cancer through modulating immune balance with low cytotoxic and effective compounds from different food and herb materials [5,6,7,8]. Recently, cancer immunotherapy has been a promising method and shines a light on prostate cancer.

Cancer immunotherapy is a method to strengthen immune system to clear tumors in vivo, including viral vaccines, cytokine therapy and cancer vaccines [9]. Characteristics of cancer immunotherapy are highly specific, efficient and durable for certain cancers; therefore, it is promising for treating prostate cancer and may be an auxiliary method to traditional surgery or radiotherapy. The ultimate goal of cancer immunotherapy is to rectify disordered immune balance induced by tumor cells in the cancer microenvironment and inhibit subsequent growth or progression of tumor cells [10]. There is growing evidence that active phytochemicals including polyphenols and flavonoids may reduce or prevent the risk for inflammation and cancer development [9]. Recently, plant-derived active ingredients from traditional herbal medicine, such as *Euodia ruticarpa,* have been found to increase an immune activity and may serve as an agent to treat prostate cancer via immunotherapy [6].

*Euodia ruticarpa* (ER)*,* also known as *Tetradium ruticarpum*, is a clinically- and widely-used in traditional medicine in East Asia and has been recognized in Korean and Japanese Kampo [11]. The fruit of *Tetradium ruticarpum* called Tetradii fructus, Evodiae fructus or Wu Zhu Yu in Chinese has been prescribed to treat several different diseases such as nausea after eating, vomiting, diarrhea, abdominal pain, headache, pelvic inflammation and dysmenorrhea for thousands of years [11]. ER has been recently found to have various biological properties targeting at anti-inflammatory and anti-cancerous potential [6,12,13,14]. ER used in Wu Chu Yu Tang has been found to have a considerable effect similar to omeprazole for treating gastroesophagel reflux disease in human in a randomized, double-blind and placebo-controlled trial [14]. Therapeutic effects of bioactive ingredients isolated from ER have been extensively studied for treating various diseases such as cancer, inflammation, central neural system, obesity, cardiovascular diseases and bacterial infection in vitro and in vivo [11]. ER and its bioactive ingredients, such as evodiamine, have been found to exert the antitumor effects on several cancer cell lines primarily through inducing cell cycle arrest, apoptosis, autophagy, inhibition of migration, invasion or angiogenesis in vitro and in vivo [11]. Rutaecarpine, that is a plant alkaloid in ER, has been evidenced to decrease prostate cancer cell growth in allogenic TRAMP-C1 prostate cancer mice and related to Th1 immune balance [8]. ER and its bioactive phytochemicals are suggested to have potential to treat prostate cancer through their potent immunomodulatory activities. Most recently, steam distilled essential oil (SDEO) isolated from ER has been proven to possess anti-inflammatory activity, suggesting that ER SDEO may have anti-cancer potential [6].

Essential oils (EOs) that are complex mixtures extracted from plants through steam distillation or various solvents have become the focus of botanicals for health promotion, including anti-parasite load in vivo [15]. Paclitaxel, that is a famous botanical compound derived from Eos, has been widely used for treating different cancers [16]. Besides, EOs have been used in aromatherapy and display different physiological functions such as anti-inflammatory activities and anti-cancer potential [17,18]. Particular EOs extracted from different parts (roots, flowers or leaves) of *Leonurus sibiricus* L., *Pallines spinose* Mentha, *Pimenta dioica*, *Rosmarinus officinalis* and *Euodia ruticarpa* (A. Juss.) Benth. have been reported to have anti-inflammatory activities [6,19,20,21,22]. Particularly, ethanol extracts of ER possess anticancer properties to inhibit benign prostatic hyperplasia-1 cell viability through caspase-8- and caspase-3-dependent apoptosis [12,23].

Medicinal plants rich in active phytochemicals are considered biologically active plants and may be an integral part of the food composition. Medicinal plants have been used to treat many diseases, including cancers, for years. Ethanol extract of *Houttuynia cordata* has been introduced in treating human colon carcinoma HT-29 cells and found to induce apoptosis through activating reactive oxygen species (ROS) production and mitochondrial membrane depolarization, increasing Bax/Bcl-2 protein ratio, forming caspase-9 protein complex, and activating caspase-3 [24]. Licorice (*Glycyrrhiza uralensis* Fisch.) polysaccharides significantly inhibit tumor growth through increasing the thymus/spleen index and the number of T lymphocytes (CD4^+^ and CD8^+^) in BALB/c CT 26 tumor-bearing mice [25]. *Verbena officinalis* water extract inhibits tumor weight and increases spleen index in H22 tumor-bearing mice [26]. The active ingredients of plant extracts, such as polyphenols, flavonoids, alkaloids and terpenoids, for cancer treatments have been studied in medicinal chemistry [27].

ER SDEO has been suggested to have anti-cancer potential due to its potent anti-inflammatory activity [6]. However, its active ingredients and potential anti-PC-3 prostate cancer effects have not been fully understood. To unravel the puzzle, ER SDEO was purified using Sephadex LH-20 gel filtration chromatography. Isolated fractions were further subjected to analyze their active ingredients with GC-MS and treat PC-3 prostate cancer cells with direct action and indirect cancer treatment using splenocyte- (SCM) and macrophage-conditioned media (MCM). T helper type 1 lymphocytes (Th1)/Th2 cytokines in SCM and pro-/anti-inflammatory cytokines in MCM were measured using enzyme-linked immunosorbent assay (ELISA). The relationships between PC-3 cell viabilities and corresponding total polyphenols, flavonoids contents as well as Th1/Th2 cytokine profiles in SCM were analyzed using the Pearson product–moment correlation coefficient (r).

## 2. Material and Methods

### 2.1. Preparation and Purification of Euodia ruticarpa (ER) Steam Distilled Essential Oil (SDEO)

Dried fruits of *Euodia ruticarpa* (A. Juss.) Benth. (www.theplantlist.org, accessed on 1 July 2021), aka *Tetradium ruticarpum* (A. Juss.) T.G. Hartley or Wu Chu Yu in Chinese, were commercially obtained by Di Hua Chinese Medicine Co. Ltd., Taichung, Taiwan (ROC). The commercial product is clinically used in prescription by Chinese medical physician and widely used by people for making tea in Taiwan. The use of Euodia fruits in the present study complies with guidelines of Taiwan government. The voucher specimen of *Tetradium ruticarpum* (A. Juss.) T.G. Hartley has been deposited in National Plant Genetic Resources Center (https://www.npgrc.tari.gov.tw, accessed on 1 July 2021), Taiwan Agricultural Research Institute Council of Agriculture, Executive Yuan. In the present study, the dried Euodia fruits (ca. 10% moisture content) were ground into a powder for extracting its steam distilled essential oil (SDEO). The SDEO extraction procedure was performed as described previously [6]. Briefly, to the sample powder (100 g) was added 10 fold deionized water to extract SDEO at 90 °C for 8 h using a rotary evaporator. The vaporized steam mixture was collected using a condensed cooler. To isolate components, the steam mixture was further extracted with a total of 400 mL ethyl acetate three times. Ethyl acetate extracts were collected and the solvent (ethyl acetate) in the mixture was removed with a rotary evaporator to obtain ER SDEO for use. ER SDEO was further purified as the following procedure. When use for cell experiments, ER SDEO and its isolated fractions were dissolved in dimethyl sulfoxide (DMSO) to prepare a 50 mM stock solution and sterilized using a filter with 0.22 μm pore size (Millipore).

To characterize ER SDEO, ER SDEO was dissolved in deionized water and appropriately diluted to detect absorption spectra scanning from 190 to 1100 nm wavelength with a spectrophotometer. ER SDEO exhibited a major absorption peak at 220 nm and a minor peak at 280 nm with a shoulder from 310 to 380 nm, suggesting that ER SDEO may rich in phenolic and flavonoid compounds (Figure S1). Thus, absorbance at 220 and 280 nm wavelengths were selected for detection when ER SDEO was purified using a gel filtration chromatography.

To purify, ER SDEO was dissolved in deionized water and adjusted to 1 mg/mL for use. An aliquot of 1 mL sample was loaded into a column (15 × 120 mm) packed with Sephadex LH-20 gel. The column was eluted subsequently with aliquots of 40 mL deionized water/methanol mixture at different ratios of 100:0, 80:20, 50:50, 20:80, 0:100 (*v/v*) and finally eluted with deionized water/acetone (20:80, *v/v*) at a flow rate of 1 mL/min, respectively. An aliquot of 4 mL eluent was collected in a tube and the absorbance of each tube was measured at 220 and 280 nm wavelength, respectively. Six isolated ER SDEO fractions (denoted as ERF1–F6, 40 mL/fraction) were obtained (Figure S2). The solvent in each isolated fraction was removed using a rotary evaporator and lyophilized.

### 2.2. Characterization of Active Ingredients in ER SDEO and Its Isolated Fractions

ER SDEO had been found to contain phytochemicals with anti-inflammatory potential such as total polyphenols (33.3 ± 0.7 mg gallic acid equivalent/g sample) and flavonoids (20.3 ± 1.6 mg quercetin equivalent/g sample) [6]. In this study, isolated ERF1–F6 were further assayed their total phenolic and flavonoid contents as previously described [6,28]. Furthermore, individual active ingredients in the isolated ERF1–F6 were analyzed using gas chromatography–mass spectrometry (GC-MS) as described previously [6].

### 2.3. Isolation of Mouse Primary Immune Cells

Female BALB/c mice (8 weeks old) were supplied by the National Applied Research Laboratories, Ministry of Science and Technology in Taipei, Taiwan. The mice were raised in the Department of Food Science and Biotechnology at National Chung Hsing University, Taichung, Taiwan, and kept on a laboratory standard diet (chow diet) and free access to drinking water. All mice were housed in a standard animal room with 12-h dark/light cycles and maintained at 23 ± 2 °C, 50–75% ambient humidity. The protocol for experimental animal use was examined and approbated (IACUC No: 103-119) by the Institutional Animal Care and Use Committee, National Chung Hsing University, Taiwan. Following adaptation for 2 weeks, the BALB/c mice (10 weeks old) were sacrificed humanely to isolate peritoneal macrophages and splenocytes [29]. The experimental mice were anaesthetized with 2% isoflurane (cat. no., 4900-1605, Panion and BF Biotech Inc., Taipei, Taiwan) using a vaporizer machine (CAS-01, Northern Vaporiser Limited, Cheshire, England, UK), bled with retro-orbital venous plexus puncture, and then sacrificed immediately with CO_2_ suffocation to isolate primary peritoneal macrophages and primary splenocytes [30]. The number of viable cells was counted with a hemocytometer using the trypan blue exclusion method. Isolated macrophages were adjusted to a cell density of 2 × 10^6^ cells/mL and isolated splenocytes were adjusted to 1 × 10^7^ cells/mL for use [6,31]. The protocol for experimental animal use was examined and approbated (IACUC No: 103-119) by the Institutional Animal Care and Use Committee, National Chung Hsing University (NCHU), Taiwan. The experiments were carried out according to the guidelines and regulations of the IACUC, NCHU. The present study is reported in accord with ARRIVE (Animal Research: Reporting of In Vivo Experiments) guidelines.

### 2.4. Effects of Treatments with ER SDEO and Its Isolated Fractions ERF1–F6 on Th1/Th2 Cytokines Secreted by Mouse Primary Splenocytes

To assess the effects of ER SDEO and its isolated fractions ERF1–F6 on Th1/Th2 cytokine secretions by splenocytes, primary splenocytes (1 × 10^7^ cells/mL, 1 mL/well) were cultured with ER SDEO and its isolated fractions ERF1–F6 at the indicated concentrations of 0, 0.5, 2, 10 and 50 μg/mL (1 mL/well) in 24-well plates, respectively. Non-cytotoxic final concentrations of 0, 0.25, 1, 5 and 25 μg/mL were achieved. Lipopolysaccharide (LPS, a B-cell mitogen, Sigma-Aldrich Co., L-2654, St. Louis, MO, USA) and concanavalin A (Con A, a T-cell mitogen, Sigma-Aldrich Co., C2010, Schnelldorf, Germany) at 2.5 μg/mL were selected as positive controls. The plates were incubated in a humidified incubator with 5% CO_2_ and 95% air at 37 °C for 48 h. The cultured plate was centrifuged at 400× *g* for 10 min. The cell culture supernatants were collected and stored at −80 °C for Th1 (IL-2)/Th2 (IL-10) cytokine assays [30,32].

### 2.5. Effects of Treatments with ER SDEO and Its Isolated Fractions ERF1–F3 on Pro-/Anti-Inflammatory Cytokines Secreted by Mouse Peritoneal Macrophages in the Absence or Presence of LPS

ER SDEO and ERF1–F3 at the indicated concentrations of 0, 0.5, 2, 10 and 50 μg/mL (500 μL/well) were cultured with mouse primary peritoneal macrophages (2 × 10^6^ cells/mL, 500 μL/well) in the absence or presence of LPS at a final concentration of 2.5 μg/mL to assess anti-inflammatory potential. Dexamethasone (DEX) at a final concentration of 100 nM was selected as a positive control against LPS-induced inflammation. The plate was incubated in a humidified incubator with 5% CO_2_ and 95% air at 37 °C for 48 h. After incubation, the supernatant in the cell culture was collected and stored at −80 °C for pro-/anti-inflammatory cytokine assays [32,33].

### 2.6. Th1/Th2 and Pro-/Anti-Inflammatory Cytokine Assays with an Enzyme-Linked Immunosorbent Assay (ELISA)

Th1 (IL-2)/Th2 (IL-10) cytokines secreted by splenocytes and pro-inflammatory (IL-1β, IL-6, and TNF-α) and anti-inflammatory (IL-10) cytokines secreted by macrophages were, respectively, measured using ELISA kits (mouse DuoSet ELISA Development system, R&D Systems, MN, USA) according to the manufacturer’s instruction. The limit of detection (LOD) of the ELISA kits used in this study was <15.6 pg/mL [30,32,33].

### 2.7. Preparation of Splenocyte-Conditioned Media (SCM) and Macrophage-Conditioned Media (MCM) with ER SDEO and Its Isolated Fractions ERF1–3

ER SDEO and its isolated fractions ERF1–F3 at the indicated concentrations of 0, 0.5, 2, 10 and 50 μg/mL (0.5 mL/well) were cultured with primary splenocytes (1 × 10^7^ cells/mL in TCM medium, 0.5 mL/well) or peritoneal macrophages (2 × 10^6^ cells/mL in TCM medium, 0.5 mL/well) in 24-well plates, respectively. The plates were incubated at 37 °C in a humidified incubator with 5% CO_2_ and 95% air for 48 h. The plate was centrifuged at 400× *g* for 10 min to collect the supernatant (1.0 mL/well) in the cell cultures (denoted as splenocyte-conditioned medium (SCM) or macrophage-conditioned medium (MCM)). The immune cell culture supernatant was collected and lyophilized. The lyophilized SCM and MCM were, respectively, dissolved in a 0.5 mL F-12K medium (GIBCO, Grand Island, NY, USA) and stored at −80 °C until use [30,32,33].

### 2.8. Culture of Human Prostate Cancer PC-3 Cells

The human prostate cancer PC-3 cell line was purchased from the Bioresource Collection and Research Center (Food Industry Research and Development Institute, Hsinchu, Taiwan, ROC) and maintained in F-12K medium supplemented with 7% fetal bovine serum (FBS, GIBCO, Grand Island, NY, USA), penicillin 100 units/mL, streptomycin 100 μg/mL, and amphotericin B 0.25 μg/mL at 37 °C in a 5% CO_2_. The PC-3 cells were adjusted and plated at a density of 2 × 10^5^ cells/mL in 96-well plates to perform the following bioassay [34].

### 2.9. Direct Treatment Effects of ER SDEO and Its Isolated Fractions ERF1–3 on the Growth of Human Prostate Cancer PC-3 Cells

The PC-3 cells (2 × 10^5^ cells/mL, 50 μL/well) were cultured in F-12K medium supplemented with 7% fetal bovine serum (GIBCO, Grand Island, NY, USA). Briefly, 50 μL aliquot of PC-3 cell suspension (2 × 10^5^ cells/mL) was treated with aliquots of 50 μL ER SDEO and its isolated ERF1–F3 fractions at the indicated concentrations of 0, 0.5, 2, 10 and 50 μg/mL in a 96-well plate to achieve final concentrations of 0, 0.25, 1, 5 and 25 μg/mL, respectively. After incubated for 24 or 48 h, the cytotoxic activity of treated samples against prostate cancer PC-3 cells was determined using 3-(4,5-dimethylthiazol-2-yl)-2,5-diphenyltetrazolium bromide (MTT) assay. Briefly, aliquots of 10 μL MTT (5 mg/mL in phosphate buffered saline (PBS)) were added to each well in a 96-well plate and incubated for another 4 h. The plate was centrifuged at 400× *g* for 10 min and the culture supernatant carefully removed. The remaining cell pellet was carefully washed twice with PBS buffer. An aliquot of 100 μL DMSO was added to each well and oscillated for 30 min to lyse the cells and dissolve formazan crystals formed in viable cells. The absorbance at 550 nm was detected with an ELISA reader (Microplate Reader FLUOstar-Omega, 415-1103, Ortenberg, Germany). The cell viability was calculated according to the relative percentage compared with the mean absorbency of the control and expressed as changes of cell numbers (% of control). Change of cell numbers (% of control) in each biological determination was calculated using the following equation: change of cell numbers (% of control) = ((A_sample_ − A_blank_)/(A_control_ − A_blank_)) × 100 [34].

### 2.10. Effects of Treatments with SCM or MCM Cultured with ER SDEO and Its Isolated Fractions ERF1–F3 on Human Prostate Cancer PC-3 Cell Growth

PC-3 cells (2 × 10^5^ cells/mL; 50 μL/well) were, respectively, treated with SCM, MCM (50 μL/well) or paclitaxel at 2.5 μΜ (as a positive control) and incubated in a humidified incubator with 5% CO_2_ and 95% air at 37 °C for 24 or 48 h. The remaining viable cells were determined by MTT assay [34].

### 2.11. Statistical Analysis

Results are presented as the mean ± SD. Differences among treatments were analyzed with one-way ANOVA, followed by DuncaN′s multiple range test using the SPSS system 20.0. Relationships between different parameters were analyzed using the Pearson product–moment correlation coefficient (r). It was considered significant if *p* < 0.05.

## 3. Results

### 3.1. Characterization of Active Ingredients in ER SDEO and Its Isolated Fractions

UV-visible absorption spectra of ER SDEO exhibited a major absorption peak at 220 nm and a minor peak at 280 nm with a shoulder from 310 to 380 nm, suggesting that ER SDEO may rich in phenolic and flavonoid compounds (Figure S1). Therefore, absorbance at 220 and 280 nm wavelengths were selected for detection when ER SDEO was purified using a Sephadex LH-20 gel filtration chromatography. Based on the chromatograms, ER SDEO further resolved into six fractions ERF1–F6 (Figure S2). The six isolated fractions were first analyzed their total polyphenol and flavonoid contents. The results showed that all of isolated fractions are rich in polyphenols and flavonoids (Table 1), identical to our assumption based on ER SDEO absorption spectra (Figure S1). Importantly, ERF1–F3 has much higher amounts of total polyphenols, suggesting that these three isolated fractions might be main active components in ER SDEO (Table 1). Active ingredients of ERF1–F6 were further subjected to analyze by GC-MS, showing that ERF1–F6, particularly in ERF1–F3, contained at least 20 compounds, including 1 alcohol, 5 acids, 3 amines, 1 ester as well as other volatile compounds (Table 2). In particular, ERF3 consisted of the greatest amount of active ingredients including palmitic acid, 2-[3-methoxyphenyl]-4H-1-benzopyran-4-one, oleic acid, 1,1-diphenyl-3-methyl-1-silacyclopent-3-ene, tetradecanoic acid, cobalt(I), cyclopentadienyl-(η^4^-cis-5,6-diethylcyclohex-1,3-diene), N,N′-diphenyl-1,4-benzenediamine, benzoic acid, N-propylbenzamide, dipropylene glycol dibenzoate, 2,2,4,5-tetramethyl-6-(1-methyloctadecyl)-1,3-dioxane and erucyl amide. Unfortunately, we found that some components repeatedly existed in different fractions (Table 2). We supposed that many factors, including the pretreatment of samples, colloidal stability, the optimal mobile phase solvent and operation time, can influence the purity of isolated fractions although the separation technology has been well developed. Importantly, identified individual active ingredient in ER SDEO isolated fractions may be provided for active candidate compounds for being studied in the future.

### 3.2. ER SDEO and ERF1–F6 Effects on Th1/Th2 Cytokine Secretions Using Mouse Primary Splenocytes

**Figure 1:**
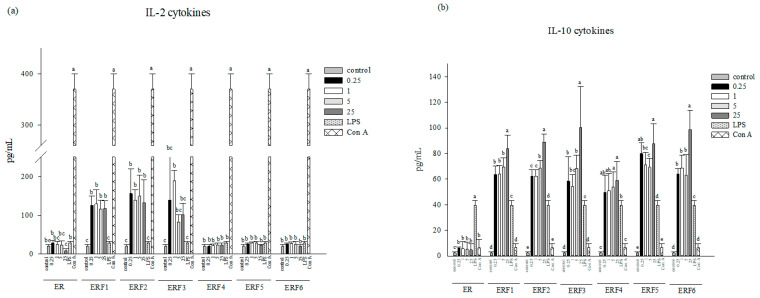

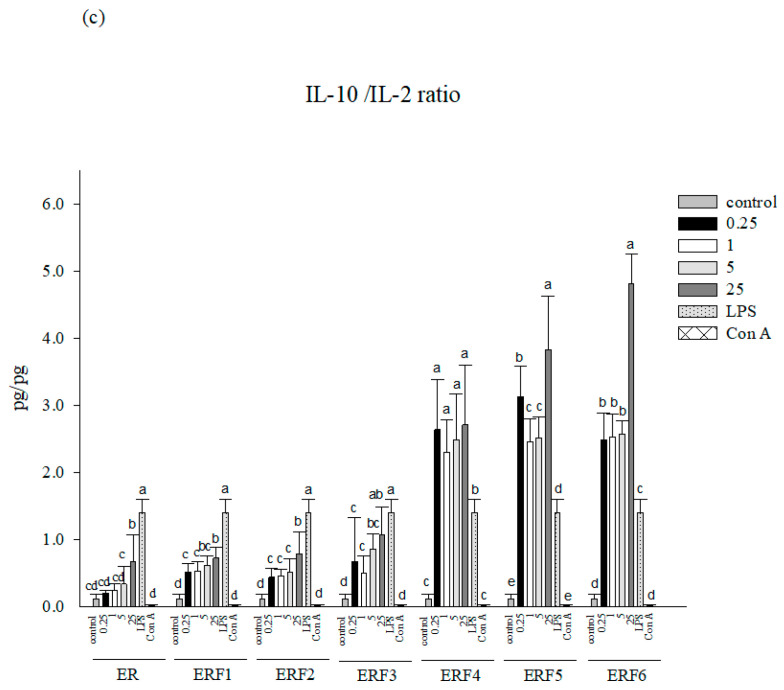
Effect of treatments with different concentrations of steam distilled essential oils of ER, and fractions 1–6 in vitro on IL-2 (**a**), IL-10 (**b**), and IL-10/IL-2 (**c**) cytokine secretions ratios by primary splenocytes from female BALB/c mice. Data are presented as the mean ± SD (*n* = 6 biological determinations). Bars in the same plot within the same sample item not sharing a common letter are significantly different (*p* < 0.05) from each other assayed by one-way ANOVA, followed by DuncaN′s multiple range test. The detection sensitivity of cytokine ELISA kits used in this study was <15.6 pg/mL.

To evaluate the effects of different ER SDEO and its isolated fractions ERF1–F6 on Th1/Th2 cytokine secretions, test samples at the indicated non-cytotoxic concentrations were, respectively, administered to splenocytes for 48 h. As shown in Figure 1, IL-10 levels in primary splenocytes cultures were significantly (*p* < 0.05) increased by ERF1–F6 compared to that of the control. It is worth noting that ERF1–F6 treatments markedly increased IL-10 (Th2 and an anti-inflammatory cytokine) secretions as compared to the treatment of ER SDEO, suggesting that ER SDEO has been appropriately purified through the gel filtration chromatography (Figure S2). Importantly, ERF1–F3, but not ERF4-F6, increased IL-2 (Th1 and a T-cell growth factor) secretions, suggesting that ERF1–F3 might have significant biological activities to T lymphocytes. Most importantly, IL-10/IL-2 (Th2/Th1) cytokine secretion ratios by splenocytes were significantly (*p* < 0.05) increased by ERF1–F6 in a dose-dependent manner, suggesting that ERF1–F6 have a Th2-inclination and anti-inflammatory property. Overall assessment indicated that only ERF1–F3 could markedly increase both IL-2 and IL-10 cytokine secretions. Meanwhile, they increased IL-10/IL-2 (Th2/Th1) cytokine secretion ratios by splenocytes dose-dependently, suggesting that ERF1–F3 is the major active fraction in ER SDEO. Our results evidenced that ERF1–F3 have immunomodulatory potential with immune stimulatory, Th2-polarized and anti-inflammatory property. Since the ERF1–F3 were the main active fractions among ER SDEO isolated fractions, the ERF1–F3 were further selected to assay their anti-inflammatory potential using mouse peritoneal macrophages.

### 3.3. ER SDEO and ERF1–F3 Effects on Pro-/Anti-Inflammatory Cytokine Secretions Using Mouse Peritoneal Macrophages in the Absence or Presence of LPS

**Figure 2:**
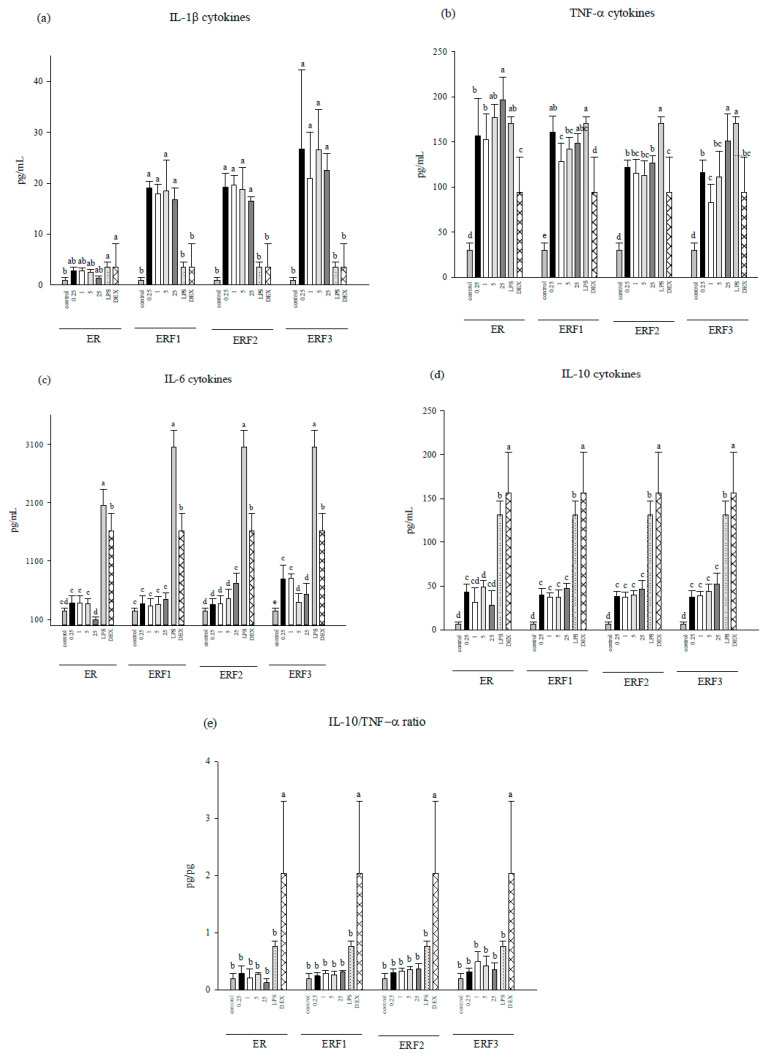
Effect of treatments with different concentrations of steam distilled essential oils of ER, and fractions 1–3 in vitro on IL-1β (**a**), TNF-α (**b**), IL-6 (**c**), IL-10 (**d**), and IL-10/TNF-α (**e**) cytokine secretions ratios by peritoneal macrophages from female BALB/c mice. Data are presented as the mean ± SD (*n* = 6 biological determinations). Bars in the same plot within the same sample item not sharing a common letter are significantly different (*p* < 0.05) from each other assayed by one-way ANOVA, followed by DuncaN′s multiple range test. Each cell population (1 × 10^6^ cells/mL medium) was, respectively, treated with the *Euodia ruticarpa* (ER) SDEO, and fractions 1–3 at the indicated concentrations of 0, 0.25, 1, 5 and 25 μg/mL. The detection sensitivity of cytokine ELISA kits used in this study was <15.6 pg/mL.

To evaluate anti-inflammatory potential, ER SDEO and ERF1–F3 at the indicated non-cytotoxic concentrations were administered to peritoneal macrophages in the absence or presence of LPS (2.5 μg/mL) for 48 h. The results showed that pro- (TNF-α, L-1β, IL-6) and anti-inflammatory (IL-10) secretions by macrophages in the absence of LPS were significantly (*p* < 0.05) increased by ERF1–F3, suggesting that ERF1–F3 have an immune stimulatory activity to macrophages (Figure 2). However, IL-10/(IL-1β+TNF-α+IL-6) (anti-/pro-inflammatory) cytokine secretion ratios slightly, but not significantly (*p* > 0.05), increased by ERF1–F3, suggesting that ERF1–F3 might have slight potential to inhibit spontaneous inflammation. In addition, IL-6 cytokines were significantly (*p* < 0.05) decreased by ER SDEO treatment at 25 μg/mL, suggesting an anti-inflammatory potential against spontaneous inflammation.

**Figure 3:**
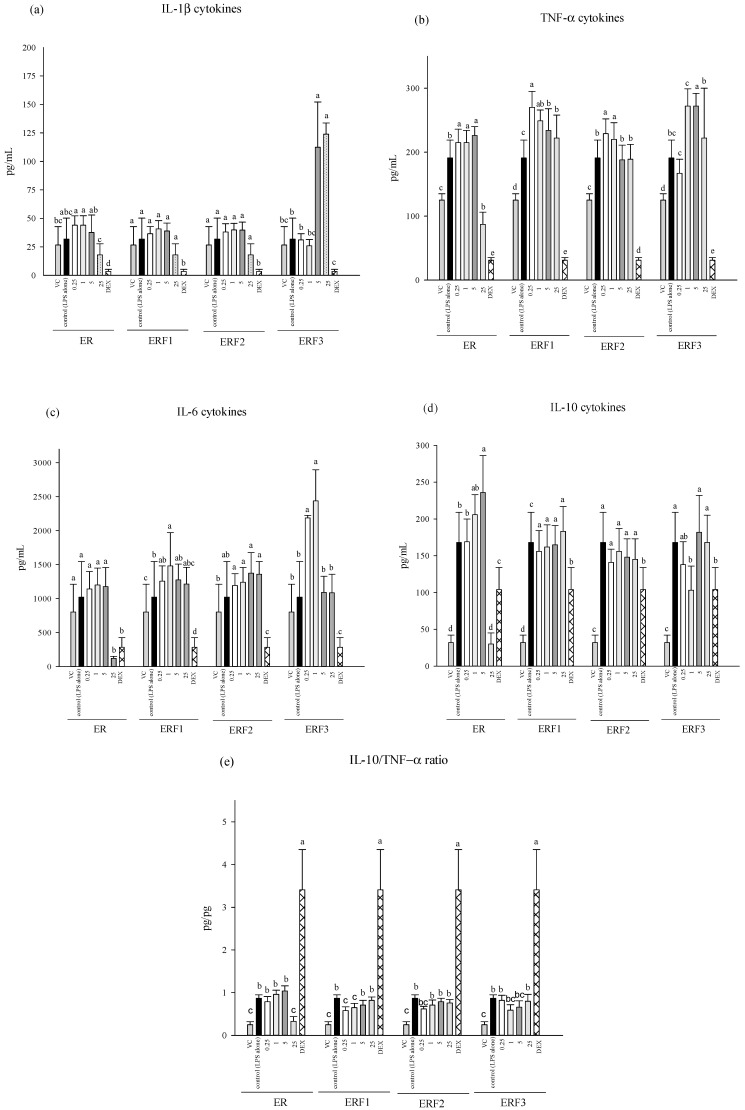
Effect of treatments with different concentrations of steam distilled essential oils of ER, and fractions 1–3 on IL-1β (**a**), TNF-α (**b**), IL-6 (**c**), IL-10 (**d**), and IL-10/TNF-α (**e**) cytokine secretions ratios by LPS-stimulated peritoneal macrophages from female BALB/c mice in vitro. Data are presented as the mean ± SD (*n* = 6 biological determinations). Bars in the same plot within the same sample item not sharing a common letter are significantly different (*p* < 0.05) from each other assayed by one-way ANOVA, followed by DuncaN′s multiple range test. Each cell population (5 × 10^6^ cells/mL medium) was, respectively, treated with the *Euodia ruticarpa* (ER) SDEO, and fractions 1–3 at the indicated concentrations of 0, 0.25, 1, 5 and 25 μg/mL. The detection sensitivity of cytokine ELISA kits used in this study was <15.6 pg/mL. VC: vehicle control.

Furthermore, ER SDEO and ERF1–F3 administration increased pro-inflammatory TNF-α, IL-6 and anti-inflammatory IL-10 cytokines using macrophages in the presence of LPS (Figure 3). However, ER SDEO and ERF1–F3 administration slightly, but not significantly (*p* > 0.05), increased IL-10/(IL-1β+TNF-α+IL-6) (anti-/pro-inflammatory) cytokine secretion ratios by LPS-stimulated macrophages. Our results suggest that ER SDEO and ERF1–F3 treatments activate LPS-induced macrophages but have mild anti-inflammatory potential. Since ER SDEO and ERF1–F3 had anti-inflammatory potential (Figure 2 and Figure 3), they were further selected to study the inhibition to prostate cancer PC-3 cell growth via either direct action or indirect treatment using splenocyte- (SCM) or macrophage-conditioned media (MCM).

### 3.4. ER SDEO and ERF1–F3 Direct Action against PC-3 Cell Growth

**Figure 4:**
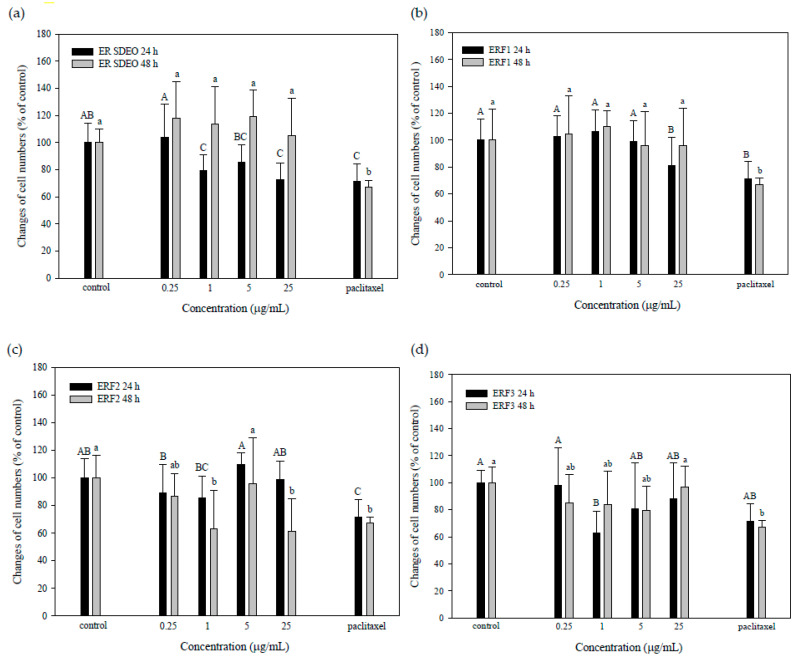
Effects of direct administrations with ER SDEO (**a**), ERF1 (**b**), ERF2 (**c**), and ERF3 (**d**) on the growth of PC-3 cells. Cells (1 × 10^6^ cells/mL) were treated for 24 and 48 h, respectively. Values are means ± SD (*n* = 6 biological determinations). Bars in the same plot at the same incubation time not sharing a common letter are significantly different (*p* < 0.05) from each other analyzed by one-way ANOVA, followed by Duncan′s multiple range test. Paclitaxel at 2.5 μM, a positive control.

To evaluate the direct action of ER SDEO and ERF1–F3 against PC-3 cell growth, ER SDEO and ERF1–F3 at the indicated concentrations of 0, 0.25, 1, 5 and 25 μg/mL were used to treat PC-3 cells for 24 or 48 h, respectively. Paclitaxel administration at 2.5 μM was selected as a positive control. Our results showed that paclitaxel at 2.5 μM significantly inhibited (*p* < 0.05) PC-3 cell growth through either 24- or 48-h incubation (Figure 4). Importantly, the growth of PC-3 cells was significantly decreased (*p* < 0.05) by ER SDEO (1 and 25 μg/mL), ERF1 (25 μg/mL), and ERF3 (1 μg/mL) as compared to the control through 24-h incubation. Through 48-h incubation, the growth of PC-3 cells was significantly decreased (*p* < 0.05) by ERF2 (1 and 25 μg/mL) as compared to the control at the same incubation time. In comparison with ER SDEO isolated fractions, ERF2 seemed to have direct long-acting effects on PC-3 cell growth. Our results evidenced that ERF1–F3 at appropriate concentrations more or less inhibit PC-3 cell growth in a direct action manner.

### 3.5. Indirect Treatment against PC-3 Cell Growth by SCM or MCM in the Absence or Presence of ER SDEO and Its Isolated Fractions ERF1–F3

**Figure 5:**
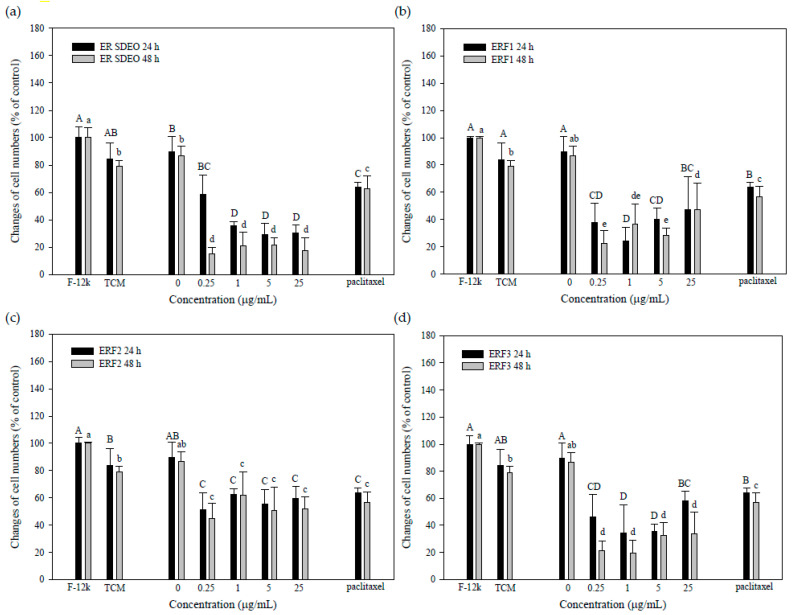
Effects of SCM prepared in the absence or presence ER SDEO (**a**), ERF1 (**b**), ERF2 (**c**), and ERF3 (**d**) on PC-3 cell growth. Cells (1 × 10^6^ cells/mL) were treated with SCM for 24 and 48 h, respectively. Values are means ± SD (*n* = 4 biological determinations). Bars in the same plot at the same incubation time not sharing a common letter are significantly different (*p* < 0.05) from each other assayed by one-way ANOVA, followed by DuncaN′s multiple range test. SCM, splenocyte-conditioned medium. PC, positive control (paclitaxel at 2.5 μΜ).

**Figure 6:**
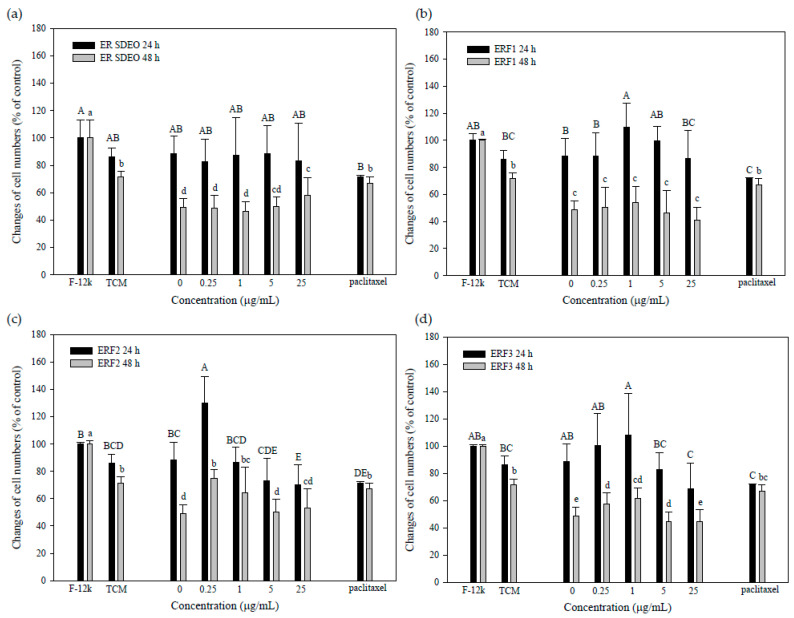
Effects of MCM prepared in the absence or presence ER SDEO (**a**), ERF1 (**b**), ERF2 (**c**), and ERF3 (**d**) on PC-3 cell growth. Cells (1 × 10^6^ cells/mL) were treated with MCM for 24 and 48 h, respectively. Values are mean ± SD (*n* = 6 biological determinations). Bars in the same plot at the same incubation time not sharing a common letter are significantly different (*p* < 0.05) from each other assayed by one-way ANOVA, followed by DuncaN′s multiple range test. MCM, macrophage-conditioned medium. PC, positive control (paclitaxel at 2.5 μΜ).

The results showed that PC-3 cell viabilities cultured in TCM media alone through 24-h or 48-h incubation were slightly decreased as compare to those in F-12K media alone, suggesting that TCM media in SCM or MCM might rather influence PC-3 cell viabilities (Figure 5 and Figure 6). Interestingly, PC-3 cells cultured in SCM without ER SDEO and its isolated fractions ERF1–F3 could not significantly (*p* > 0.05) influence PC-3 cell growth (Figure 5). Most importantly, viabilities of PC-3 cells cultured in SCM with ER SDEO and its isolated fractions ERF1–F3 through either 24- or 48-h incubation were significantly (*p* < 0.05) decreased (Figure 5). SCM cultured with ER SDEO and its isolated fractions ERF1–F3 exhibited the better inhibitory effect on PC-3 cell viability than paclitaxel administration at 2.5 μM through either 24- or 48-h incubation (Figure 5). Our results suggest that tumor treatment against prostate cancer PC-3 cells using splenocytes with main active fractions from ER SDEO might have the better inhibitory effect than that of chemotherapy using paclitaxel (Figure 5). Our results further suggest that components in ER SDEO and its isolated fractions ERF1–F3 markedly changed splenocytes’ secretion profiles, e.g., Th1/Th2 cytokine ratios. In the present study, we have evidenced that ERF1–F3 markedly increased both IL-2 and IL-10 cytokine secretions; meanwhile, they dose-dependently raised IL-10/IL-2 (Th2/Th1) cytokine secretion ratios by splenocytes, indicating that ERF1–F3 have immune stimulatory, Th2-polarized and anti-inflammatory properties (Figure 1). We hypothesized that the tumor microenvironment with immune stimulatory, Th2-polarized and anti-inflammatory cytokines may effectively inhibit the PC-3 cell viability.

In addition, the viabilities of PC-3 cells incubated for 48 h were significantly (*p* < 0.05) inhibited by MCM in the absence of ER SDEO and its isolated fractions ERF1–F3 (Figure 6). Unfortunately, the inhibitory effect against PC-3 cell viabilities for 48-h incubation treated with MCM in the presence of ER SDEO and its isolated fractions ERF1–F3 could not significantly (*p* > 0.05) enhanced. Identical to our hypothesis, our results suggested that ERF1–F3 might just have slight potential to inhibit spontaneous inflammation (Figure 2), resulting in that they cannot markedly influence the tumor microenvironment via changing macrophages’ secretion profiles. Compared with the effects between SCM and MCM, SCM (possibly T and B cells), but not MCM, has dominant effects on PC-3 cell viabilities. Therefore, SCM was further selected to analyze its association with other different parameters.

### 3.6. Associations between Th2 (IL-10)/Th1 (IL-2) Cytokine Secretion Levels in Primary Splenocyte Cultures Treated with ER SDEO as Well as Its Isolated Fractions ERF1–F3 and Total Polyphenol and Flavonoid Contents in Their Corresponding SCM Media

**Figure 7:**
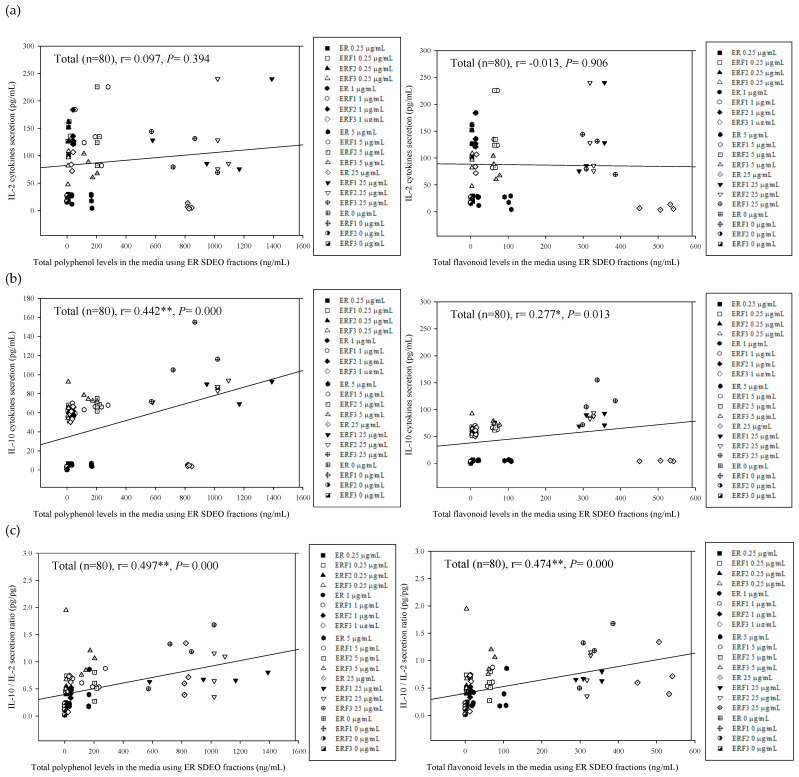
The associations between total polyphenol and flavonoid contents in the media using ER SDEO as well as ERF1–F3 and IL-2 (**a**), IL-10 (**b**), as well as IL-10/IL-2 cytokine secretion ratios (**c**) by splenocytes. The correlation was expressed by Pearson product–moment correlation coefficient (r). * *p* < 0.05; ** *p* < 0.01.

To clarify the relationship between cytokine secretion profiles by splenocytes and active ingredients in their corresponding media, correlations between total polyphenol and flavonoid contents in ER SDEO and its isolated fractions ERF1–F3 in the SCM and cytokine secretion levels in the cultures were determined using the Pearson product–moment correlation coefficient (r) (Figure 7). The results showed that total polyphenol and flavonoid contents in the media were positively correlated with IL-10 (Th2) (*r* = 0.442, ** *p* = 0.000; *r* = 0.277, * *p* = 0.013) as well as IL-10/IL-2 (Th2/Th1) cytokine secretion ratios (*r* = 0.497, ** *p* = 0.000; *r* = 0.474, ** *p* = 0.000) by splenocytes through 24-h and 48-h incubation, respectively (Figure 7a–c). Our results evidenced that active ERF1–F3 phytochemicals, particularly total polyphenols and flavonoids, markedly increased Th2 (IL-10) cytokines, suggesting that active phytochemicals in SDEO such as phenolic and flavonoid compounds may have Th2-polarized and anti-inflammatory property through changing Th1/Th2 cytokine secretion profile.

### 3.7. Associations between PC-3 Cell Viabilities Treated with SCM and Cytokine Secretion Levels in Their Corresponding SCM

**Figure 8:**
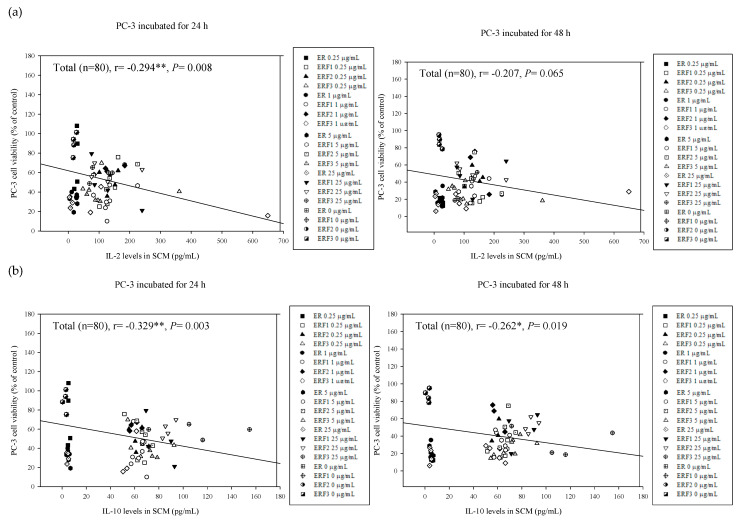

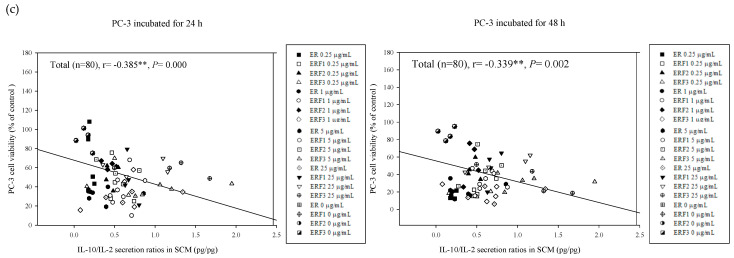
The associations between IL-2 (**a**), IL-10 (**b**), as well as IL-10/IL-2 cytokine secretion ratios (**c**) in SCM and their corresponding PC-3 cell viabilities treated with SCM for 24 and 48 h, respectively. The correlation was expressed by Pearson product–moment correlation coefficient (r). * *p* < 0.05; ** *p* < 0.01.

To clarify the relationship between cytokine secretion profiles in SCM and viabilities of PC-3 cells treated with SCM without or with ER SDEO and its isolated fractions ERE1-F3, the correlations were determined using the Pearson product–moment correlation coefficient (r) (Figure 8). We found that IL-2 (Th1) cytokine secretion levels in the SCM were significantly negatively correlated with PC-3 cell viabilities (*r* = −0.294, ** *p* = 0.008) through 24-h incubation (Figure 8a). IL-2 is a well-known T cell growth factor that can promote T cell proliferation. Our results indicate that increased IL-2 levels may inhibit the PC-3 cell viability. IL-2 may be developed for treating prostate cancer in the future. Unfortunately, the significant correlation vanishes through 48-h incubation (Figure 8a), possibly resulting from the short half-life of most cytokines. In addition, IL-10 (Th2 cytokine) and IL-10/IL-2 (Th2/Th1) secretion ratios in the SCM are significantly negatively correlated with PC-3 cell viabilities through 24-h (*r* = −0.329, ** *p* = 0.003; *r* = −0.385, ** *p* = 0.000) and 48-h incubation (*r* = −0.262, * *p* = 0.019; *r* = −0.339, ** *p* = 0.002), respectively (Figure 8b,c). Most importantly, our results suggest that an anti-inflammatory (IL-10) cytokine and Th2-polarized immune balance in the tumor microenvironment may inhibit PC-3 cell growth. The underlining of increased IL-2 and IL-10 cytokines is important in inhibiting PC-3 cell viability. Solid clues have been provided in the present study; however, more evidence concerning this phenomenon in vivo should be accumulated for application to treat the prostate cancer using indirect immunotherapy in the future.

### 3.8. Associations between PC-3 Cell Viability Treated with Direct Action and Total Polyphenol and Flavonoid Contents in ER SDEO and Its Isolated Fractions ERF1–F3

**Figure 9:**
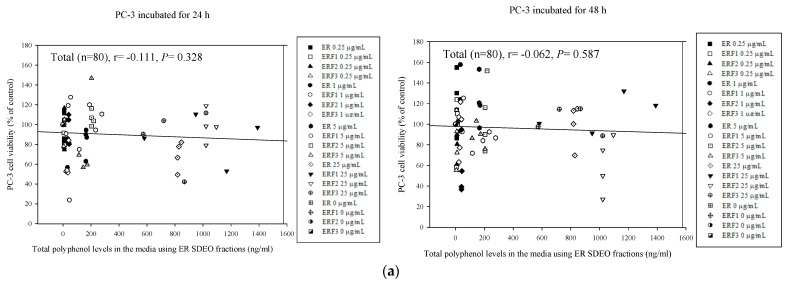

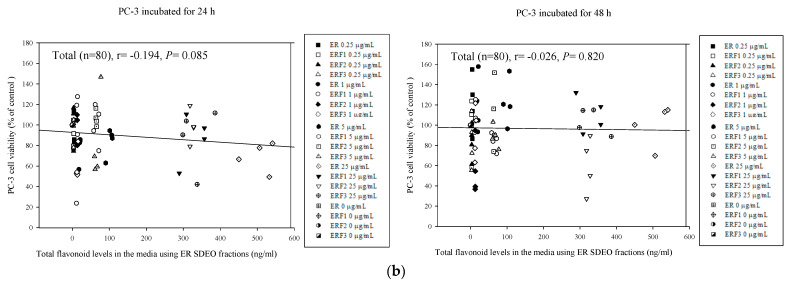
The associations between total polyphenol (**a**) as well as flavonoid contents (**b**) in the media and their corresponding PC-3 cell viabilities treated with SDEO samples for 24 and 48 h, respectively. The correlation was expressed by Pearson product–moment correlation coefficient (r).

Correlations between total polyphenol and flavonoid contents in the media with ER SDEO as well as its isolated fractions ERF1–F3 and PC-3 cell viabilities were determined using the Pearson product–moment correlation coefficient (r) (Figure 9). The results showed that total polyphenol and flavonoid contents in the media were slightly, but not significantly *(p* > 0.05), negatively correlated with PC-3 cell viability through either 24-h or 48-h incubation, reflecting that total polyphenol and flavonoid in SDEO purified fractions might play a minor role in the inhibition to PC-3 cell viability with direct action (Figure 9). In comparison with these associations, it can be concluded that phenolics and flavonoids may effectively modulate the immune balance and subsequently inhibit PC-3 cell growth, rather than directly inhibiting PC-3 cell growth.

## 4. Discussion

Recently, diverse essential oils have been studied for treating cancers. Lavender essential oil as well, as its active composition linalool, and linalyl acetate have been found to inhibit PC-3 cell growth through cell cycle arrest in the G2/M phase, inducing subsequent apoptosis [2]. Cold pressing essential oil of *Gannan navel* orange peel decreases the proliferation of a prostate cancer cell line 22RV-1 [35]. *Cinnamomum cassia* essential oil was found to induce G2/M arrest in the cell cycle, apoptotic characteristics of the observed DNA laddering, chromatin condensation and cytochrome c release in the mitochondria [36]. *Pinus koraiensis* essential oil was found to be active against proliferation and migration of HCT116 cells [37]. The activity of essential oils against different cancers is mainly attributed to their active ingredients through the synergic or antagonist action. Because of the lesser side effects than chemotherapy drugs, natural active ingredients in essential oils may have great potential to treat advanced or metastatic prostate cancer. In the present study, we evidenced that active ingredients of ERF1–F3 from *Euodia ruticarpa* steam distilled essential oil (ER SDEO) have potential to inhibit the PC-3 prostate cancer through indirect treatment with immune cells conditioned media (Figure 5), possibly attributing by their high amounts of total polyphenols (Table 1). To date, at least 165 chemical components have been identified from ER, including steroids, terpenoids, phenolic acids, flavonoids, alkaloids and phenylpropanoids [11]. In the present study, GC-MS analysis suggests that active ingredients such as 2-[3-methoxyphenyl]-4H-1-benzopyran-4-one and N,N′-diphenyl-1,4-benzenediamine in ERF1, 2-[3-methoxyphenyl]-4H-1-benzopyran-4-one and N,N′-diphenyl-1,4-benzenediamine in ERF2, and 2-[3-methoxyphenyl]-4H-1-benzopyran-4-one, N,N′-diphenyl-1,4-benzenediamine, benzoic acid, N-propylbenzamide and dipropylene glycol dibenzoate in ERF3 which are the diverse derivate of phenolic compounds are suggested to be potent anti-PC-3 cancer agents (Table 2). However, the anti-PC-3 cell viability effects by each individual phenolic derivate should be further verified in the future.

The physiological activity of essential oils is mainly attributed to their active ingredients through synergistic or antagonistic effects. So far, steroids, terpenes, phenolic acids and alkaloids have been identified from *Euodia ruticarpa* [11]. In the present study further analysis of the active ingredients of ERF1–F6 by GC-MS showed that ER SDEO contained alcohols, acids, amines, esters and other volatile compounds (Table 2). Although derivatives of phenolic compounds may be considered effective for anti-PC-3 cancer drugs. Unfortunately, most of phenolic compounds are still poorly understood and cannot be quantified. In addition to focusing on the advantages of polyphenols, we still need to pay attention to their possible adverse side effects, cytotoxic effects at the higher dosage used in the experiment and the limit of bioavailability in vivo. Recently, it has been reported that there has been an in vitro additive effect of resveratrol (a polyphenol) at the higher concentrations on astrocyte swelling if post-exposure with ammonia, ischemia and trauma [38]. Polyphenols are not always good for health; therefore, we should be prudent when phenolic compounds are isolated from different sources for use. Although there is limited relevant current research pointing out phenolics’ toxicity and safe dosage, inappropriate doses may be poisonous. This requires further long-term research to verify the anti-PC-3 effect of each phenol derivative, although the dose effects on the cell viability in vitro may provide some information to understand their positive and negative effects on prostate cancer growth (Figure 4). Based on our results, it is suggested that lower concentrations of active ingredients may avoid their adverse side effects.

Moreover, medicinal plants rich in flavonoids and polyphenolic compounds have been widely used for different purposes, particularly anticancer, in the world [39]. Polyphenols as active ingredients are well-known antioxidants, but they also play a role in promoting oxidation. Certain polyphenols, such as isoflavones, may have carcinogenic and genotoxic effects, which interfere with thyroid hormone biosynthesis, nonheme iron absorption and certain pharmaceutical agents to enhance their biologic effects [40]. It needs extensive research efforts to ascertain the optimal dosage of polyphenol compounds in various foods or medicinal plants for safe use and to avoid their potential harmful side effects [41]. Even though there are many beneficial effects on health, some polyphenol compounds at high dosage are found to be pro-oxidant or mutagenic with toxicity [41]. It is found that treatments of cultured astrocytes with the higher concentration of resveratrol (>25 µM) increase ammonia, ischemia, as well as trauma-induced cell swelling through increasing phosphorylated ERK1/2 and p38MAPK, as well as enhancing the activity of the Na^+^-K^+^-Cl^−^ co-transporter-1 (NKCC1), further suggesting a lower concentration at 5 or 10 µM of resveratrol for a protective and safe dose [38]. Although protective effects of polyphenols are suggested for use in different acute and chronic diseases, potential harmful effects of particular polyphenols still should be considered and counterbalanced with current limited evidence of harm based on the research literature [42]. The food intake is usually lower than the dose used in the study, therefore the daily diet may not cause the occurrence of disease [39,43,44]. To date, international governmental regulations have been discussed and officially sanctioned only a few specific polyphenolic compounds for their safety and health claims [42]. Dietary supplements rich in polyphenol can potentially provide additional benefits, but high-doses may cause critical toxicity [45]. We still need to pay attention to the hidden worries caused by these active ingredients.

Active ingredients in ER SDEO isolated fractions rich in total polyphenols and flavonoids exerted their anticancer capability to PC-3 cells via regulating Th1 (IL-2) and Th2 (IL-10) cytokine production by splenocytes with indirect treatment of conditioned media (Figure 7 and Figure 8). The spleen is the largest immune organ in the human body, comprised mainly of 47% T cells and 42% B cells, as well as a small quantity of other antigen presenting cells, possibly reflecting the entire immune balance in vivo [46]. The synergy of IL-2 and IL-10 cytokines advances the CD8^+^ T cell cytotoxicity effect [47]. Consistent with other researches, our results suggest that active ingredients of ER SDEO and its isolated fractions ERF1–F3 may enhance anti-cancer immunity through increasing T cells or activating NK cells and B cells [48,49]. We hypothesized that the synergistic effect between different components in fractions may offset the weakness of an individual component (Table 2). The present study has shown that SCM and MCM (especially SCM) inhibited PC-3 cell growth through cytokine treatment (Figure 5 and Figure 6). The SCM used in the research can reflect the status of the adaptive immune system [46]. Our results further evidence that T lymphocytes and B lymphocytes may be the key immune cells to inhibit the growth of PC-3 cells [46]. PC-3 cells are non-hormonal dependent human prostate cancer cells, lacking androgen receptor and prostate-specific antigen [50], resulting in a poor treatment effect with hormone therapy in the later stage. Natural anti-cancer constituents in Chinese herbal medicine have been isolated for clinical trials, particularly via their potential heat-clearing, detoxicating and immunomodulatory effects [51,52]. Recently, rutaecarpine, which is a main alkaloid in *Euodia ruticarpa,* has been evidenced to inhibit cancer cell growth in an allogenic TRAMP-C1 prostate cancer mouse model through amending damaged immune balance in vivo [8]. The present study further provides an alternative method for treating non-hormonal dependent human prostate cancers with cytokine treatment using ER SDEO and its active fractions ERF1–F3 in an indirect manner via immune cells conditioned media. This study is just an in vitro study; therefore, more in vivo studies should be tested in the future. In general, active ingredients of herbal plant extracts are mainly tannins, flavonoids, alkaloids, polyphenols and terpenoids. Most recently, we have found that an alkaloid rutaecarpine in *Euodia ruticarpa* inhibited the growth of prostate cancer in mice [8]. Thus, we expect active constituents isolated from ER SDEO may also have potential to inhibit PC-3 prostate cancer cell growth in vivo.

Essential oils rich in volatile active compounds extracted from different parts of various plants may be used for aromatherapy through the skin and respiratory tract to directly act on the respiratory, circulatory and central nervous system [53]. Aromatherapy may be often used as an auxiliary treatment to reduce the anxiety of cancer patients, relieve pain or improve the quality of life. It seems to have lower side effects than many traditional anti-cancer drugs and it is obviously worthy of further clinical application and scientific discussion. In the present study, we found that ER SDEO may modulate cytokine secretion profiles, particularly IL-2 and IL-10. In clinical practice, IL-2 has been proven to be a promising method to prevent allograft rejection and treat autoimmune and inflammatory diseases [54]. IL-2 is a cytokine approved by the US Food and Drug Administration for the treatment of cancer [55]. The purified active fractions of ER SDEO increased the secretion of cytokines IL-2 and IL-10 by mouse primary splenocytes from female BALB/c mice in the present study. So far, few studies have found to increase the secretions of both Th1 (IL-2) and Th2 (IL-10) cytokines at the same time, meaning that the purified active fractions can wholly enhance immunity.

## 5. Conclusions

In the present study, ER SDEO resolved into six ERF1–F6 fractions using Sephadex LH-20 gel filtration chromatography detected at 220 and 280 nm wavelengths. ERF1–F3 fractions were rich in total polyphenols and flavonoids with diverse active ingredients. ERF1–F3 significantly increased IL-10 secretions and IL-10/IL-2 secretion ratios by splenocytes. IL-1β, TNF-α, IL-6 and IL-10 secreted by macrophages were markedly increased by ER SDEO and ERF1–F3. Meanwhile, IL-10/TNF-α secretion ratios were slightly increased, suggesting that ERF1–F3 have immunostimulatory, Th2-polarized and anti-inflammatory characteristics. Our results evidenced that ERF1–F3 at appropriate concentrations more or less inhibits PC-3 cell growth in a direct action manner. SCM and MCM treatments alone significantly inhibited PC-3 cell growth, suggesting that cytokine secretions by splenocytes and macrophages, particularly splenocytes, may involve in inhibiting PC-3 cell growth. SCM cultured with ER SDEO and ERF1–F3 markedly enhanced their inhibitory effects on PC-3 cell growth. There are significantly negative correlations between PC-3 cell viabilities and IL-2, IL-10 as well as IL-10/IL-2 ratios in SCM. Total polyphenol and flavonoid contents in the media were positively correlated with IL-10 (Th2) as well as IL-10/IL-2 (Th2/Th1) cytokine secretion ratios by splenocytes through 24-h and 48-h incubation, respectively. Our results suggest that the inhibitory effects on PC-3 prostate cancer cells may be attributed to active anti-inflammatory ingredients in ER SDEO and its active ERF1–F3 fractions via a direct action manner and modulating splenocytes’ cytokine secretion profiles. Active ingredients in ER SDEO and its active ERF1–F3 fractions may be used as an alternative agent for food and medicine to treat PC-3 prostate cancers in the future.

## Figures and Tables

**Table 1:** Contents of total flavonoid and polyphenol levels in ER fractions 1–6.

Samples	Total Flavonoids (mg Quercetin Equivalent/g Sample)	Total Polyphenols (mg Gallic Acid Equivalent/g Sample)
ERF1	13.1 ± 1.4 ^a,b^	40.9 ± 13.8 ^a^
ERF2	12.9 ± 0.2 ^a,b^	41.6 ± 1.5 ^a^
ERF3	13.3 ± 1.6 ^a,b^	31.8 ± 17.7 ^a,b^
ERF4	14.4 ± 0.7 ^a^	10.7 ± 1.5 ^b,c^
ERF5	12.4 ± 0.8 ^b,c^	4.8 ± 1.4 ^c^
ERF6	11.4 ± 0.5 ^c^	24.0 ± 10.6 ^b^

Data are presented as the mean ± SD (*n* = 4 replications). Values within same column not sharing a common superscript small letter are significantly different (*p* < 0.05) from each other analyzed using one-way ANOVA, followed by DuncaN′s multiple range test.

**Table 2:** Chemical components of ER fractions 1–6 assayed with Gas Chromatography–Mass Spectrometry (GC–MS).

Fractions	NO.	RT (min)	RI	Compounds	M.W.	Chemical Formula	CAS NO.
**F1**	1	33.510	1536.44	1-Dodecanethiol	202.18	C_12_H_26_S	000112-55-0
	2	47.825	2089.37	2-[3-Methoxyphenyl]-4H-1-benzopyran-4-one	252.08	C_16_H_12_O_3_	007622-32-4
	3	53.663	2364.47	Cobalt(I), cyclopentadienyl-(η^4^-cis-5,6-diethylcyclohex-1,3-diene)	260.10	C_15_H_21_CO	000000-00-0
	4	53.721	2367.58	N,N′-Diphenyl-1,4-benzenediamine	260.13	C_18_H_16_N_2_	000074-31-7
	5	54.934	2427.84	Dehydroabietic acid	300.21	C_20_H_28_O_2_	001740-19-8
	6	62.832	2779.08	Erucylamide	337.33	C_22_H_43_NO	000112-84-5
**F2**	1	47.823	2089.37	2-[3-Methoxyphenyl]-4H-1-benzopyran-4-one	252.08	C_16_H_12_O_3_	053906-83-5
	2	53.658	2364.47	Cobalt(I), cyclopentadienyl-(η^4^-cis-5,6-diethylcyclohex-1,3-diene)	260.10	C_15_H_21_Co	000000-00-0
	3	53.716	2367.58	N,N′-Diphenyl-1,4-benzenediamine	260.13	C_18_H_16_N_2_	000074-31-7
	4	62.781	2775.81	9-Octadecenamide	281.27	C_18_H_35_NO	000301-02-0
**F3**	1	44.625	1952.63	Palmitic acid	256.24	C_16_H_32_O_2_	000057-10-3
	2	47.823	2089.37	2-[3-Methoxyphenyl]-4H-1-benzopyran-4-one	252.08	C_16_H_12_O_3_	053906-83-5
	3	48.701	2129.13	Oleic acid	282.26	C_18_H_34_O_2_	000112-80-1
	4	49.833	2180.61	1,1-Diphenyl-3-methyl-1-silacyclopent-3-ene	250.12	C_17_H_18_Si	051343-48-7
	5	53.371	2350.27	Tetradecanoic acid	256.24	C_16_H_32_O_2_	000124-06-1
	6	53.659	2364.47	Cobalt(I), cyclopentadienyl-(η^4^-4-cis-5,6-diethylcyclohex-1,3-diene)	260.10	C_15_H_21_CO	000000-00-0
	7	53.718	2367.58	N,N′-Diphenyl-1,4-benzenediamine	260.13	C_18_H_16_N_2_	000074-31-7
	8	55.087	2435.53	Benzoic acid	314.12	C_18_H_18_O_5_	000120-55-8
	9	55.214	2441.89	N-Propylbenzamide	163.10	C_10_H_13_NO	010546-70-0
	10	55.701	2466.14	Dipropylene glycol dibenzoate	342.15	C_20_H_22_O_5_	020109-39-1
	11	55.902	2476.09	2,2,4,5-Tetramethyl-6-(1-methyloctadecyl)-1,3-dioxane	410.41	C_27_H_54_O_2_	056324-82-4
	12	62.861	2779.08	Erucylamide	337.33	C_22_H_43_NO	000112-84-5
**F4**	1	44.607	1952.63	Palmitic acid	256.24	C_16_H_32_O_2_	000057-10-3
	2	55.893	2476.09	2,2,4,5-Tetramethyl-6-(1-methyloctadecyl)-1,3-dioxane	410.41	C_27_H_54_O_2_	056324-82-4
	3	57.294	2540.93	2-Propenoic acid, 2-ethylhexyl ester	184.15	C_11_H_20_O_2_	000103-11-7
	4	62.335	2775.81	9-Octadecenamide	281.27	C_18_H_35_NO	000301-02-0
	5	62.865	2779.08	Erucylamide	337.33	C_22_H_43_NO	000112-84-5
**F5**	1	62.724	2775.81	9-Octadecenamide	281.27	C_18_H_35_NO	000301-02-0
**F6**	1	47.825	2089.08	2,4-Dibromo-phenol	249.86	C_6_H_4_OB_r2_	000615-58-7
	2	49.475	2164.05	1,3-Bis(4-methylphenyl)-1,3-propanedione	252.12	C_17_H_16_O_2_	003594-36-3
	3	49.833	2180.61	1,1-Diphenyl-3-methyl-1-silacyclopent-3-ene	250.12	C_17_H_18_Si	051343-48-7
	4	53.669	2364.44	Titanium	260.10	C_16_H_20_Ti	000000-00-0
	5	53.721	2367.58	N,N′-Diphenyl-1,4-benzenediamine	260.13	C_18_H_16_N_2_	000074-31-7
	6	57.299	2540.50	4-(6-Methoxy-2-quinolyl)-benzonitrile	260.10	C_17_H_12_N_2_O	000000-00-0
	7	62.865	2779.08	Erucylamide	337.33	C_22_H_43_NO	000112-84-5

RT: Retention Time (min); RI: Kovats retention index for performing research data into the composition of essential oils using GC-MS; CAS NO: Chemical Abstracts Service Number.

## Data Availability

The datasets used and/or analyzed during the current study available from the corresponding author on reasonable request.

## Supplementary Materials

The following are available online at https://www.mdpi.com/article/10.3390/cimb43020071/s1, Figure S1: UV-visible absorption spectra of ER steam distilled essential oil (SDEO) using a spectrophotometer scanning from 190 nm to 500 nm. Figure S2: ER SDEO chromatograms detected by absorbance at 220 nm (a) and 280 nm (b) using a Sephadex LH-20 gel filtration chromatography.

## Abbreviations

ADT	androgen deprivation therapy
Con A	concanavalin A
CRPC	castration-resistant prostate cancer
DEX	dexamethasone
DMSO	dimethyl sulfoxide
ELISA	enzyme-linked immuno-sorbent assay
ER	*Euodia ruticarpa*
ERF1-F6	ER SDEO fraction 1–6
GC-MS	gas chromatography–mass spectrometry
IL	interleukin
LPS	lipopolysaccharide
MCM	macrophage-conditioned medium
MTT	3-(4,5-dimethylthiazol-2-yl)-2,5-diphenyltetrazolium bromide
SCM	splenocyte-conditioned medium
SDEO	steam distilled essential oil
Th1	T helper type 1 lymphocyte
Th2	T helper type 2 lymphocyte
TNF	tumor necrosis factor
