# Supplementary material for: Active Ingredients from Euodia ruticarpa Steam Distilled Essential Oil Inhibit PC-3 Prostate Cancer Cell Growth via Direct Action and Indirect Immune Cells Conditioned Media In Vitro
Source: Curr Issues Mol Biol. 2021 Aug 28;43(2):996–1018. doi: 10.3390/cimb43020071 (PMC8928987; doi:10.3390/cimb43020071)
Supplement: Supplementary file 1 [file cimb-43-00071-s001.zip › cimb-1304395-SI.pdf]

Supplementary Material

Figure S1: UV-visible absorption spectra of ER steam distilled essential oil (SDEO) using a spectrophotometer scanning from 190 nm to 500 nm.

Figure S2: ER SDEO chromatograms detected by absorbance at 220 nm (a) and 280 nm (b) using a Sephadex LH-20 gel filtration chromatography.

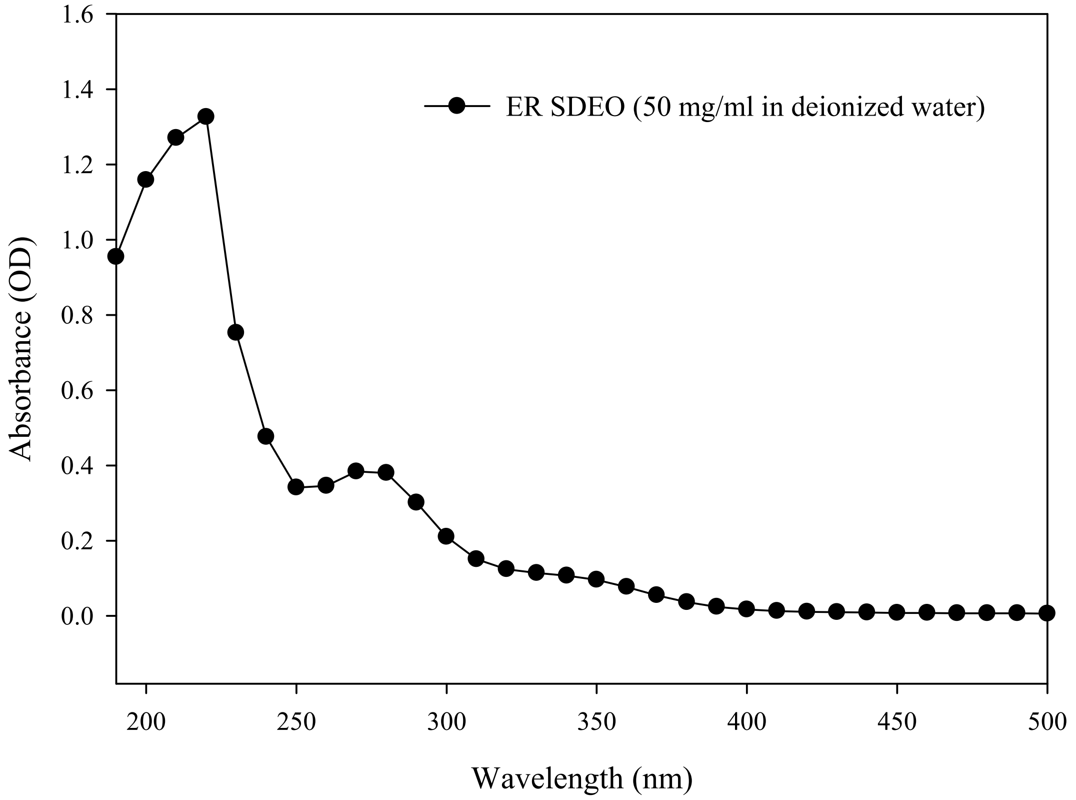

Figure S1: UV-visible absorption spectra of ER steam distilled essential oil (SDEO) using a spectrophotometer scanning from 190 nm to 500 nm.

(a)

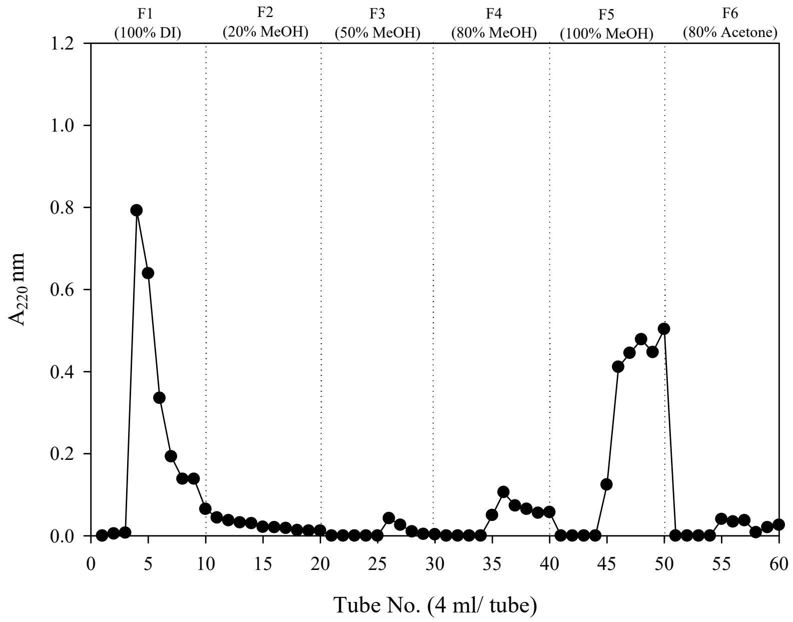

(b)

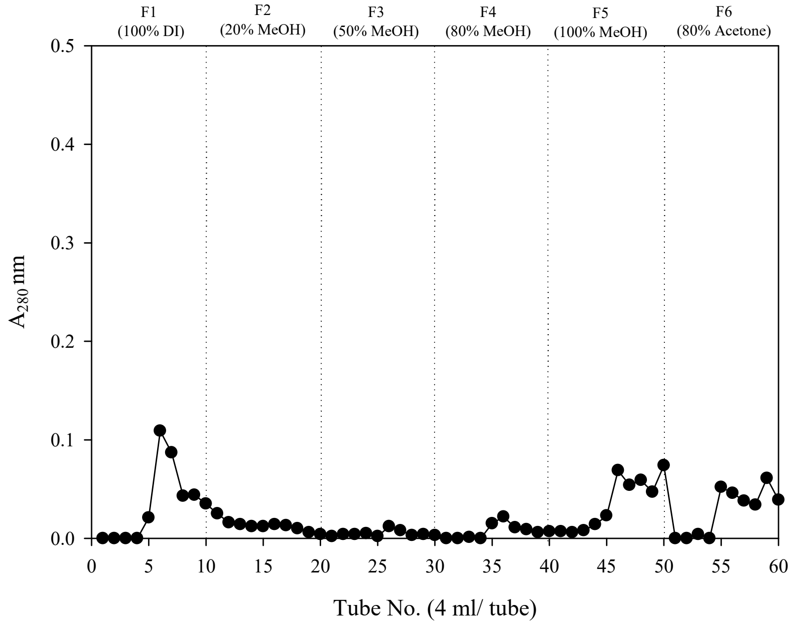

Figure S2: ER SDEO chromatograms detected by absorbance at 220 nm (a) and 280 nm (b) using a Sephadex LH-20 gel filtration chromatography.
